# EEG Microstate Features as an Automatic Recognition Model of High-Density Epileptic EEG Using Support Vector Machine

**DOI:** 10.3390/brainsci12121731

**Published:** 2022-12-17

**Authors:** Li Yang, Jiaxiu He, Ding Liu, Wen Zheng, Zhi Song

**Affiliations:** Department of Epilepsy Centre and Neurology, The Third Xiangya Hospital, Central South University, Changsha 410000, China

**Keywords:** epilepsy, EEG microstate, EEG features, SVM classifier

## Abstract

Epilepsy is one of the most serious nervous system diseases; it can be diagnosed accurately by video electroencephalogram. In this study, we analyzed microstate epileptic electroencephalogram (EEG) to aid in the diagnosis and identification of epilepsy. We recruited patients with focal epilepsy and healthy participants from the Third Xiangya Hospital and recorded their resting EEG data. In this study, the EEG data were analyzed by microstate analysis, and the support vector machine (SVM) classifier was used for automatic epileptic EEG classification based on features of the EEG microstate series, including microstate parameters (duration, occurrence, and coverage), linear features (median, second quartile, mean, kurtosis, and skewness) and non-linear features (Petrosian fractal dimension, approximate entropy, sample entropy, fuzzy entropy, and Lempel–Ziv complexity). In the gamma sub-band, the microstate parameters as a model were the best for interictal epilepsy recognition, with an accuracy of 87.18%, recall of 70.59%, and an area under the curve of 94.52%. There was a recognition effect of interictal epilepsy through the features extracted from the EEG microstate, which varied within the 4~45 Hz band with an accuracy of 79.55%. Based on the SVM classifier, microstate parameters and EEG features can be effectively used to classify epileptic EEG, and microstate parameters can better classify epileptic EEG compared with EEG features.

## 1. Introduction

Epilepsy is one of the most common neural diseases, with recurrent, persistent, and episodic characteristics [1]. Epilepsy is transient, stereotyped, recurrent, and sudden. Abnormal electrical activity in the brain causes short-lived seizures [2]. According to the International League Against Epilepsy (ILAE), epilepsy can be diagnosed as two or more seizures within an interval of 24 h. Epilepsy is a state of abnormal neural activity, which is caused by the hypersynchronous discharge of neurons. At this time, neurons show extremely active discharge activity, which induces seizures, including a loss of consciousness, tics, etc. Epilepsy affects 8% of the world’s population, both directly and indirectly [2], and patients with epilepsy (PWEs) rarely realize or predict the onset of a seizure before it occurs, which increases the risk of physical harm. The early diagnosis and subsequent evaluation of epilepsy is crucial. Due to delays in epilepsy diagnosis and untimely and inappropriate treatments, many patients develop serious complications, such as cognitive disorders, emotional disorders, etc. During a seizure, ictal epileptiform discharges (IEDs) are produced, which is a necessary condition for the diagnosis of epilepsy [3]. However, a short-term interval electroencephalogram (EEG) cannot accurately record the IEDs; therefore, the diagnosis of PWEs is based on video electroencephalogram (VEEG) in clinical practice. When interictal, electrical activity in the brains of PWEs remains relatively normal, usually without epileptiform discharges, which adds difficulty to the diagnosis of epilepsy [4]. Moreover, long-term EEG is relatively expensive, and the required labor is relatively high. Therefore, if short-term EEG can be used for the early diagnosis of epilepsy, it will greatly reduce the burden on doctors and patients. With technological developments, the number of electrodes used in clinical EEG technology has gradually developed, from the 16-lead conventional EEG to 512-lead EEG. With the greater number of electrodes, more information can be recorded by the EEG, and the more likely the doctor is to make a diagnosis. Whether low-lead or high-lead EEG is used, there will be noise during recording, such as noise caused by muscle movement [5], eye blinks [6], etc. Therefore, it is necessary to remove the noise through processing. In this study, we used microstate analysis to classify and diagnose epilepsy through short-term EEG recorded by a 128-lead EEG instrument.

To study changes in the nervous system of the brain, EEG signals provide valuable information through the oscillatory dynamics of brain waves. EEG generally refers to scalp EEG, which is used to record the electric potential of the entire scalp generated by electrical activity in the brain, and there are advantages of high temporal resolution non-invasiveness [7]. Previous studies have shown that EEG can be used as a potential biomarker to diagnose various neuropsychiatric disorders, such as schizophrenia [5], depression [7], epilepsy [8], emotional disorders [9], etc. Compared with traditional time–frequency domain analysis methods, microstate analysis, representing the current state of brain topological activity, has been studied with some success.

EEG microstates are short-lived quasi-stable electrical activity patterns in the cortex that suggest the presence of neural networks [10]. EEG microstate analysis began in 1987 with the work of Lehman et al. [11]. It indicates that the scalp potential topography is in a relatively stable state within 60~120 ms, and then quickly switches to another state and remains stable. The semi-stable nature is referred to as the EEG microstate [11]. EEG microstate is a common method used to study large-scale neural networks, which combines the temporal and spatial information of EEG, enabling the observation of millisecond-level changes in EEG topography. Lehman found that the topology of the scalp voltage topography of the resting-state EEG did not change continuously or randomly over time. Following Lehman, some researchers found that the topology of the scalp voltage topography remained stable for a certain period of time, and was then quickly converted into another scalp voltage topography and remained stable for a certain period of time, in which the intensity of the scalp voltage topography was variable [12], but the topology was constant. There are four common map shapes of microstates, named A, B, C, and D, which indicate changes in the activity of brain neurons and reflect different neurological functions of the brain [13]. These four microstates are related to four known functional systems, where each system is activated by specific cognitive functions and sensory inputs. Quantitative determination of the onset and duration of microstates can yield information related to neuronal electrical activity, which can be used for neurological-disease-related analysis [9,14]. In 2021, a study by Annamaria Painold et al. suggested that changes in brain nerve activity and functional deficits may lead to changes in EEG microstates [15].

Compared with other EEG analysis methods, microstate-based EEG analysis has several advantages. The most important of these is that the EEG microstate topographic map can be defined at any data point independently, enabling the observation of millisecond-level changes in electrical topographic maps [16]. Therefore, EEG microstates are more suitable for the rapid analysis of the dynamics of large-scale EEG activity. Microstate EEG analysis simultaneously integrates EEG signals from all electrodes to create a global functional representation that reflects the overall functional state of the brain [12]. Therefore, EEG microstate analysis represents an effective, low-cost, and maneuverable electrophysiology method for studying large-scale neural networks [17].

EEG microstate analysis can be used to detect rapid, dynamic activity in large-scale neuro-networks. Therefore, this study incorporated the microstate analysis of epileptic high-lead EEG to accurately extract the spatial and temporal information of epileptic EEG. In this study, the microstate analysis of high-lead EEG during the interictal period of epilepsy was carried out in order to obtain a model that can effectively classify the interictal EEG of PWEs and assist in the diagnosis of epilepsy.

## 2. Materials and Methods

**Figure 1:**
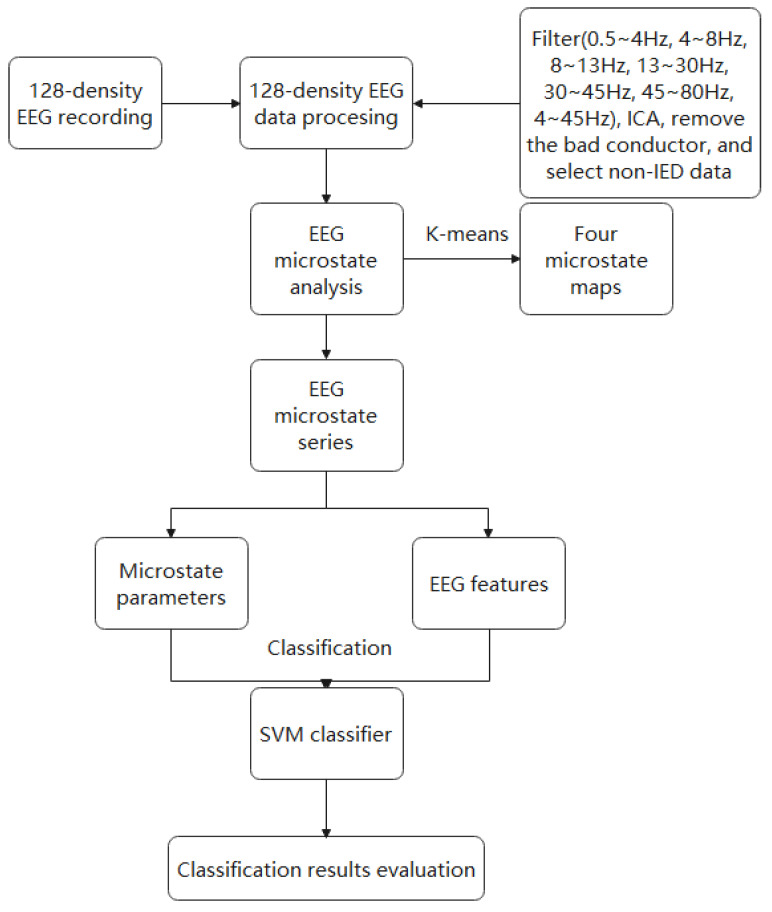
The technology roadmap of this paper.

This study was an EEG classification experiment based on EEG microstate analysis. It was based on the support vector machine (SVM) classifier, which was used to compare the effects of different feature extraction methods on EEG classification. The technology roadmap is shown in Figure 1.

### 2.1. Subjects and EEG Recording and Preprocessing

This was a single-center research study, and it passed the ethical review of the Third Xiangya Hospital (ID: 22187). EEG recordings were obtained from 32 PWEs and 20 healthy subjects, who were recruited from the Third Xiangya Hospital. The inclusion and exclusion criteria were as follows. Inclusion criteria: (1) diagnosed with focal epilepsy by two professional doctors according to the 2017-ILAE standard; (2) age ≥ 15 years or head circumference matched with electrode cap. Exclusion criteria: (1) history of other brain-related conditions (trauma, infection, etc.); (2) unable to complete EEG tasks independently; (3) unable to consent to EEG examination. Each participant received a 128-lead EEG examination with the GSN system, whose sampling rate was 1000 Hz, and the duration was 5 min. For each instance, 5 EEG values, with a duration of 45 s, were extracted to minimize noise and artifacts.

For the measurement data which showed a normal distribution, t-tests were selected for statistical analysis, and for the measurement data that did not fulfil the normal distribution and the count data, Mann–Whitney U nonparametric tests were selected for the statistical analysis.

We preprocessed all data through EEGLAB (2020b), a plug-in for MATLAB (vR2020b), to remove artifacts and noise, including filtering (0.5~4 Hz, 4~8 Hz, 8~13 Hz, 13~30 Hz, 30~45 Hz, 45~80 Hz, and 4~45 Hz), ICA, removal of the bad conductors, and the selection of non-IED data. Due to interference and the influence of patients, such as a sudden seizure or hyperactivity symptoms, which caused a large amount of artifacts that could not be removed, we finally obtained 135 epileptic EEG data values and 83 healthy EEG data values, whose duration was 45 s.

### 2.2. Microstate Analysis

**Figure 2:**
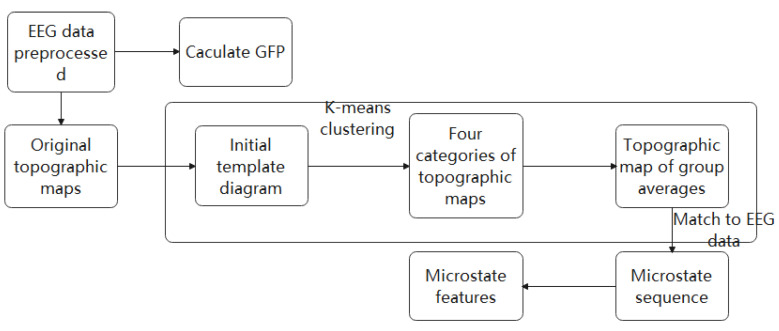
Flow chart of EEG microstate analysis. Each EEG datum point was used to calculate the GFP curve of each data point. We plotted the potential of all electrodes at the time of local maximum of the GFP curve to generate the original topographic map. The original maps were subjected to the clustering algorithm, which grouped submitted maps into a group of clusters (4 maps) according to the terrain similarity. Finally, the clustering map was inversely fitted to the GFP curve, and each data point was marked with the most relevant clustering map. Therefore, EEG signals were converted into EEG microstate sequences.

For microstate analysis, we followed the standard steps for microstate segmentation proposed by Murray et al. We used MATLAB (vR2020b) and the EEGLAB toolbox to carry out the analysis. First, we calculated the field strength at each moment (the global field power (GFP)). GFP is defined as

$$GFP=\sqrt{\frac{1}{n}\overset{n}{\underset{i=1}{\sum }}{\left(v_{i}\left(t\right)-\bar{v\left(t\right)}\right)}^{2}}$$

where v(t) is the electrode voltage vector (united as µv) at time *t*, *n* is the number of electrodes, and $v_{i}\left(t\right)$ represents the voltage of the ith electrode. In topographic maps with distinct or many peaks, when the GFP is high, the signal-to-noise ratio tends to be high; when the GFP is low, the signal-to-noise ratio tends to be low. We selected the topographic map at the peak of the GFP as the original topographic map to describe the surrounding EEG signals, which can effectively reduce the redundancy of EEG signals and the computational load. Four types of microstate topographic maps were selected as the original model topographic maps through cross-validation [13]. Subsequently, each original topographic map was spatially clustered to obtain the final model topographic map, in order to maximize the similarity between EEG samples and their designated microstate prototypes. In this study, we applied K-means spatial clustering. The flow chart of EEG microstate analysis is shown in Figure 2.

#### 2.2.1. K-Means Clustering

**Figure 3:**
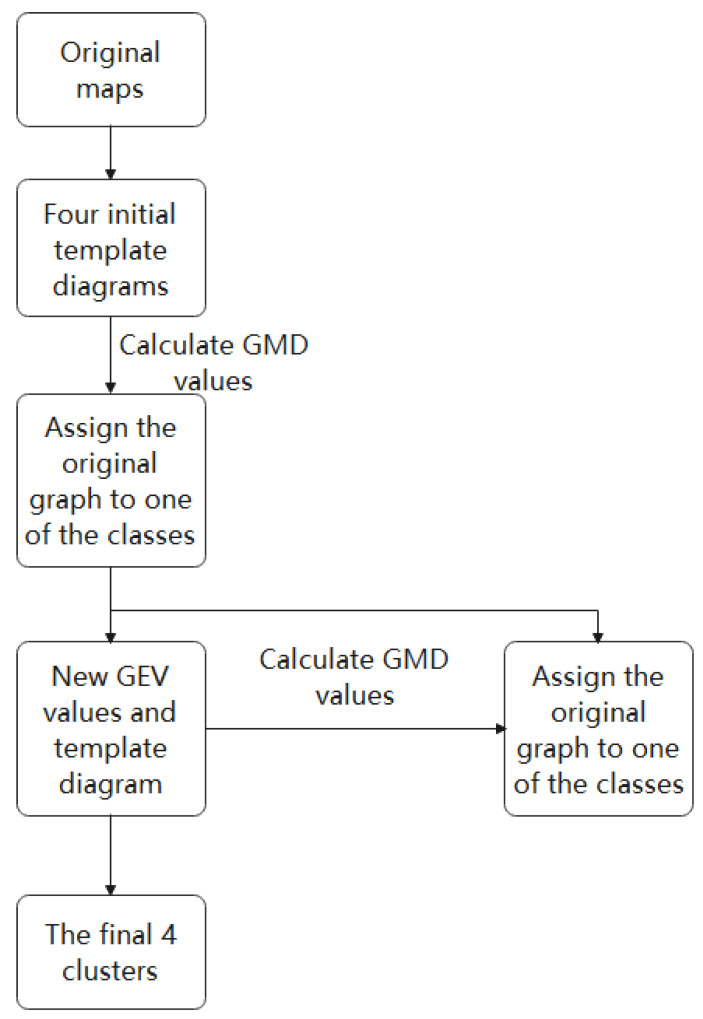
Microstate category recognition process based on K-means clustering.

In this study, we adopted the spatial clustering algorithm based on K-means clustering, as shown in Figure 3. The GMD value provided an electric-field-independent metric that describes the topological difference between two electric field topographic maps. The GMD value is defined as

$$GMD=\sqrt{\frac{1}{n}\overset{N}{\underset{i=1}{\sum }}\left\{\frac{u_{i}-\bar{u}}{\sqrt{\sum_{I=1}^{N}\frac{{\left(u_{i}-\bar{u}\right)}^{2}}{N}}}-\frac{v_{i}-v}{\sqrt{\sum_{I=1}^{N}\frac{{\left(v_{i}-\bar{v}\right)}^{2}}{N}}}\right\}}$$

In the formula, $u_{i}$ represents the voltage value of the first topographic map, $v_{i}$ represents the voltage value of the second topographic map, and N refers to the number of electrodes. The range of GMD values is from 0 to 2. Zero means that the two maps are completely consistent, and two represents the polarities of the two maps on the contrary. The algorithm of K-means clustering comes from the research of Koening et al. [12]. For each model topographic map, we will obtain a time series of spatial correlation coefficients, and for each original topographic map, we can obtain a model topographic map figure with the lowest GMD. Based on these results, we calculated the overall proportion of variance explained (GEV, which describes the proportion of variance explained by the model topographic map over all original topographic maps) for the four selected model topographic maps. We matched the model topographic map with the original topographic map based on the GMD value, marked the original map as the type with the smallest GMD, and superimposed all the original topographic maps marked with the same model topographic map to obtain four new topographic patterns. (4) We repeated step 3 until the GEV value did not increase (i.e., the GEV reached the highest level), and the final four-mode topographic map is obtained. The original scalp potential topography obtained by each subject at each time was compared and matched with the microstate (pattern topographic map) obtained by clustering, the four microstates were marked as A, B, C, and D, and the subjects were obtained. The corresponding EEG microstate time series parameters were calculated. In the EEG microstate analysis, we extracted microstate parameters and EEG signal features, and we used them as models to classify epileptic EEG using the SVM classifier.

#### 2.2.2. EEG Microstate Parameters

The basic temporal dynamics of microstates are described by occurrence, duration and coverage. The occurrence rate reflects the average times per second dominated by microstates. The duration is defined as the average duration (in milliseconds) of a given microstate. The coverage rate reflects the fraction of time for which a given microstate is active. These fractions are directly extracted through the algorithm in EEGLAB. In this study, the parameters (occurrence, duration, and coverage) were used as a model to classify epileptic EEG signals.

### 2.3. Feature Extraction

EEG signals are nonlinear; therefore, nonlinear features are commonly extracted to accurately classify signals. In this study, five types of linear features and five types of nonlinear features were extracted from the EEG microstate in the sub-band of 4~45, which is usually used in EEG analysis [18]. The linear features were the mean, second quartile, median, skewness, and kurtosis. The nonlinear features were the Petrosian fractal dimension (PFD), Lempel–Ziv complexity (LZC), and entropies (approximate entropy, sample entropy and fuzzy entropy).

#### 2.3.1. Linear Feature Extraction

Some time–frequency features were used in signal processing in the time–frequency domain. These are governed by the equations given in Table 1.

#### 2.3.2. Nonlinear Feature Extraction

##### PFD

The Petrosian fractal dimension (PFD) is a chaotic algorithm used to calculate EEG signal complexity [19]. The PFD quickly computes fractal dimensions by converting signals into binary sequences. It is governed by the following equation:

$$PFD=\frac{\log_{10}k}{\left[\log_{10}k+\log_{10}\left(\frac{k}{k+0.4N_{\delta }}\right)\right]}$$

Here, k denotes the number of signals, and $N_{\delta }$ denotes the number of changes in the signal.

##### LZC

Based on the binary coarse-grained algorithm, we calculated the mean and threshold of LZC, compared the amplitude and threshold, and converted the original signal into a 0–1 sequence [20]. Then, the number of distinct patterns in the sequence was calculated from the complexity of the signal. It was governed by the following equation:

$$LZC=\frac{c\left(n\right)}{b\left(n\right)}$$

where b(n)=nlogin, *n* is the length of the time series and *i* is the coarse-grained degree of the time series.

##### Entropy

Entropy measures the chaos within a system and is useful in measuring the ambiguity and variability in signals. In this study, four entropies were studied, including the approximate entropy (ApEn) [21], sample entropy (SampEn) [22] and fuzzy entropy [23].

ApEn is defined as

$$ApEn\left(m,r,N\right)=\Phi^{m}\left(r\right)-\Phi^{m+1}\left(r\right)$$

where $\Phi^{m}\left(r\right)={\left(N-m+1\right)}^{-1}\overset{N-m+1}{\underset{I=1}{\sum }}\ln\frac{1}{N-m+1}\overset{N-m+1}{\underset{j=1}{\sum }}\Theta \left(d_{ij}^{m}-r\right)\left(r\right)$, *N* is the length of the time series, *r* is the similarity capacity, and *m* is the embedded dimension.

SampEn is defined as

$$SampEn\left(r,m,N\right)=-\ln\frac{A^{m}\left(r\right)}{B^{m}\left(r\right)}$$

where $A^{m}\left(r\right)={\left(N-m-1\right)}^{-1}\overset{N-m-1}{\underset{I=1}{\sum }}C_{i}^{m+1}\left(r\right)$, $C_{i}^{m+1}\left(r\right)$ is the probability that the distance between the vector $X_{m}\left(j\right)$ and the vector $X_{m}\left(i\right)$ is less than r, *N* is the length of time series, *r* is the similarity capacity, and *m* is the embedded dimension.

Fuzzy entropy is defined as

$$FuzzyEn\left(m,r,N\right)=\ln\Phi^{m}\left(r\right)-\ln\Phi^{m+1}\left(r\right)$$

where $\ln\Phi^{m}\left(r\right)=\frac{1}{N-M+1}\overset{N-m+1}{\underset{i=1}{\sum }}\frac{1}{N-m}\overset{N-m+1}{\underset{j=1,i\neq j}{\sum }}A_{ij}^{m}$, *N* is the length of the time series, *r* is the similarity capacity, and *m* is the embedded dimension.

### 2.4. Training/Test Set Split

To avoid overfitting, we used cross-validation for the classification experiments. However, the data were amplified from a limited number of patients; therefore, specific data segmentation was required to prevent the classifier from learning the pattern of each patient. To this end, we randomly selected one PWE and one healthy person; for each participant, we included five data values with the same labels as the test set. The data of the other 26 PWE and 17 healthy participants (225 subjects in total) were applied in the cross-validation experiment as a training set.

### 2.5. EEG Signal Classification

SVM is a binary classification model. It can be divided into a linear model and nonlinear models according to the type of input data [24]. In our study, the EEG data were divided into a testing set with 1 epileptic EEG datum point and 1 heathy EEG datum point, and a training set. Then, we chose leave-one-out cross-validation to complete the classification task of epileptic EEG [25,26,27].

The extracted microstate parameter set and feature set were input into the SVM classifier separately. SVM has good generalization capacities to prevent overfitting. Compared with other classifiers, SVM has high robustness and generality to non-stationary signals and is widely used in machine learning analysis.

### 2.6. Evaluation of Classifier

The SVM classifier belongs to the second type of classifier, and its classification results are “Yes” and “No”. We defined the EEG of epileptic patients as “positive EEG” and the EEG of healthy subjects as “negative EEG”. We used accuracy, recall, and specificity to evaluate the classification efficiency of the SVM classifier for epileptic EEG in this study. *TP* (true positive) is the amount of positive EEG data predicted by the model as positive samples; *FP* (false positive) is the amount of negative EEG data predicted by the model as positive samples; *FN* (false negative) is defined as the amount of positive data predicted by the model as negative samples; and *TN* (true negative) is the amount of negative EEG data predicted by the model as negative samples. Then, we calculated the accuracy, recall, and specificity according to *TP*, *FP, FN*, and *TN*.

Accuracy=(TP+TN)/(TP+TN+FP+FN)

Recall=TP/(TP+FN)

Specificity=TN/(TN+FP)

The receiver operating characteristic (ROC) curve is a plot with recall on the y-axis and specificity on the x-axis. The area under the ROC curve (AUC) is a measure of the model performance. For practical situations, an AUC of over 70% is desirable [28].

## 3. Results

### 3.1. Participants’ Information

Our study recruited 44 participants, including 27 PWEs and 17 healthy participants. Their details are presented in Table 2. The clinical details of the PWEs are shown in Table 3. There was no significant difference in age, gender, and education level between PWEs and healthy participants (*p* > 0.05), as shown in Table 2.

In this section, the classification results of epilepsy and healthy states, in the absence of IEDs, from 128-lead EEG data are presented based on the EEG microstate parameters and the features of EEG signals.

**Figure 4:**
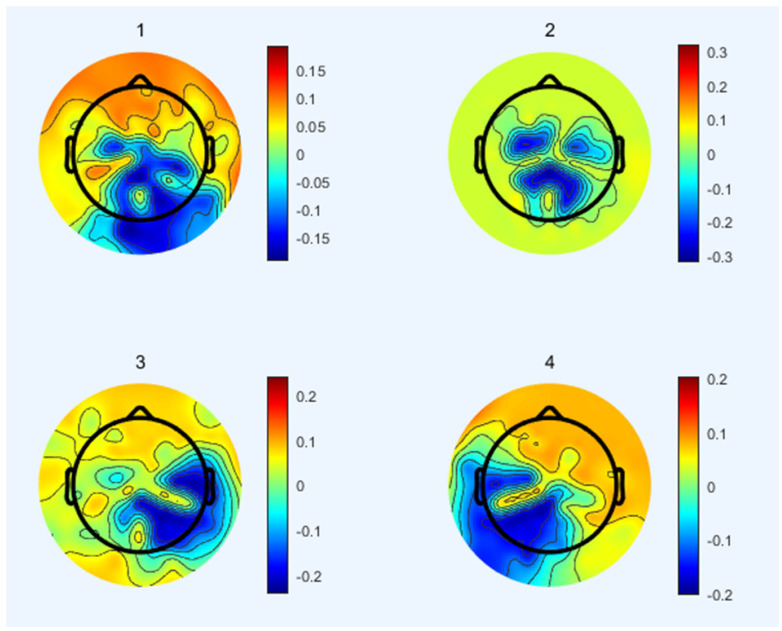
Topographies of the selected global microstate classes retrieved from the clustering algorithm for sub−bands.

Microstate maps across all participants from all sub-bands are shown in Figure 4. To identify any resemblance in the topographies from different sub-bands, topographic analysis was performed to compare microstate maps among sub-bands. No significant difference was observed in any microstate map in any sub-band.

### 3.2. EEG Microstates Parameters’ Classification

**Figure 5:**
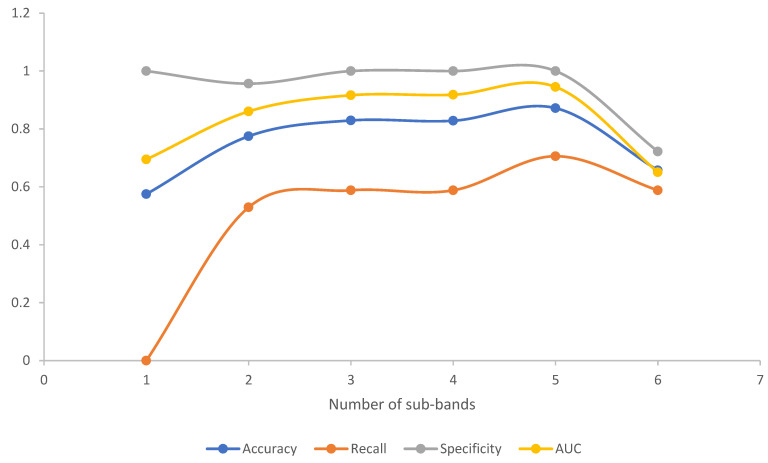
Line charts of classification results of EEG microstate parameters in different sub-bands, in which the number of sub-bands on the x-axis is the same as in Table 4. Four criteria were used to evaluate the classifier model. Obviously, the classification effect of the classifier model in the γ-band was better than in other sub-bands.

In this section, the classification of EEG microstate parameters is presented. Table 4 shows the performance of EEG microstate parameters in the SVM classifier. The results of three evaluation indexes (accuracy, recall, and specificity), at different EEG frequency sub-bands, are given. There were differences in performance in the classification of epilepsy when using the EEG features extracted from different frequency bands. The classification results of different sub-bands through SVM are shown in Table 4 and Figure 5. It is observed that the EEG microstate parameters are more suitable for the EEG classification of epilepsy in these sub-bands (α-band, β-band, and γ-band), with an accuracy of 87.18%, recall of 70.59%, and specificity of 100.00%. Furthermore, the results show that the δ-band and high band (45~80 Hz) do not contain important data for microstate analysis, because the accuracy, recall, specificity, and AUC are much lower than those of other sub-bands.

**Figure 6:**
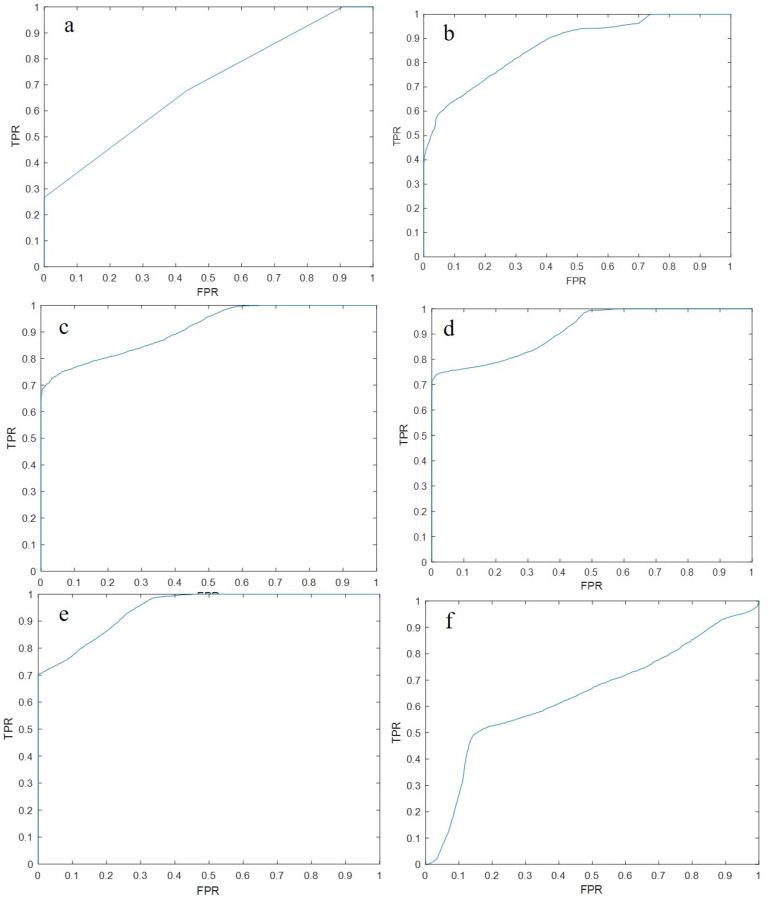
The ROC curve of epilepsy classification using the SVM classifier using microstate parameters as feature inputs, plotted with recall as same as the true positive rate (TPR) on the y-axis, and specificity as the same as the false positive rate (FPR) on the x-axis. (**a**) The ROC curve of the EEG microstate parameters in the delta (0.5~4 Hz) frequency band using the SVM classifier; (**b**) The EEG microstate parameters in the θ (4~8 Hz) frequency band using SVM. The ROC curve of the classifier classification; (**c**) The ROC curve of the α (8~13 Hz) frequency band EEG microstate parameters using the SVM classifier; (**d**) The β (13~30 Hz) curve. The ROC curve of the EEG microstate parameters of the frequency band classified by the SVM classifier; (**e**) The ROC curve of the EEG microstate parameters of the γ (30~45 Hz) frequency band classified by the SVM classifier; (**f**) The ROC curve of the EEG microstate parameters in the 45~80 Hz frequency band using the SVM classifier.

The ROC curves for different sub-bands are shown in Figure 6. Comparing the AUC in different frequency bands, we found that the EEG microstate parameters in the gamma frequency band had the best effect on subject classification, because the AUC of the gamma band was higher than that of other sub-bands for the microstate parameters. Moreover, it was found that the alpha band and beta band performed relatively well. The results are presented in Table 4.

### 3.3. EEG Feature Set Classification

In this section, the classification of EEG signal features that were extracted from the EEG microstate time series for each single channel in 4~45 Hz is presented. Table 5 shows the performance of the EEG single features in the SVM classifier. The results for accuracy, recall and specificity are presented. We can conclude that the SVM classifiers which use the feature set that included the median, mean, second quartile, kurtosis, skewness, fuzzy entropy, PFD approximate entropy, sample entropy, and LZC could effectively classify epileptic EEG signals, with an accuracy of 79.55%, recall of 81.84%, and specificity of 76.47%.

## 4. Discussion

In this study, we analyzed the epileptic EEG microstate sequence, and we could intelligently classify epileptic EEG through the features of the microstate sequence. We analyzed the EEG microstates of 27 epileptic EEG signals and 17 healthy EEG signals. Based on the microstate analysis, we extracted the microstate parameters and microstate sequence feature set and input them into the SVM classifier to classify the epileptic EEG signals. Based on the SVM classifier, the microstate parameters (duration, occurrence, and coverage) with an accuracy of 87.18% and the feature set (median, mean, second quartile, kurtosis, skewness, fuzzy entropy, PFD approximate entropy, sample entropy, and LZC) with an accuracy of 79.55%, extracted from the EEG microstate sequence with an accuracy of 79.55%, could be used as a classification model to classify epileptic EEG signals.

In 2018, Kiran et al. used machine learning to classify the EEG microstate parameters of the EEG of PWEs, and the classification accuracy rate could reach more than 76.1%; they also described the microstate changes of intractable epilepsy [8]. The results suggest that large-scale EEG microstate changes exist in PWEs, and this change can be used as a bioelectrical marker for the intelligent identification of epilepsy [23]. In this study, based on machine learning, the SVM classifier used the EEG microstate parameters as a classification model to classify the interictal EEG signals of epileptic patients and the resting EEG signals of healthy subjects. We found that in the γ (30~45 Hz) frequency band, the classification model was the most accurate for the epilepsy group and the heathy group, reaching 87.18%. This result is inconsistent with the results of Ahmedi et al. [23] on the classification of epilepsy EEG signals and psychogenic non-epileptic interictal EEG; their results showed that β (13~30 Hz) had the best classification effect. In this study, we selected patients with focal epilepsy as the experimental group; Ahmedi et al. [23] did not clearly define the epilepsy categories, and the seizure mechanism of PWEs with different parts or categories was inconsistent, which may have led to differences in the results of the two studies. These findings [8,23] demonstrate that we can efficiently classify epilepsy and healthy patients’ short-range EEG signals using machine learning algorithms based on EEG microstate parameters in the absence of abnormal epilepsy discharges.

EEG signals have linear and nonlinear characteristics [20,29]. In recent years, various models have been proposed for different EEG signal data, and feature extraction and feature classification are frequently used in various diseases, such as the diagnosis and monitoring of neuropsychiatric diseases [30]. Based on the amplitude-integrated electroencephalography (aEEG) and compress spectrum array (CDSA) theory, Abend et al. [31] performed the EEG identification of epileptic seizures; the sensitivity of identifying long-term epileptic discharges was 88%, whereas the sensitivity of identifying short-term epileptic discharges was 40% [31]. The researchers [32] then used the short-term index to classify EEG signals, revealing pathological brain electrical activity, and achieved 99.6% accuracy in the classification of healthy and pathological EEG. This means that EEG signatures can effectively identify disease EEG signals. In this study, we also combined the high temporal resolution of EEG microstates to extract the EEG signal features of the EEG microstate time series and input them into an SVM classifier to classify PWEs versus healthy subjects. Compared with previous research using epileptic interictal EEG as a subject, we obtained a better model with relatively high classification accuracy, and we chose the short-term interictal EEG signal as a research object to make the model more universal. However, compared with previous research [23,33] using EEG during the ictal stage, there was a poor classification effect and low accuracy achieved by the classification model in this study. Following an analysis of previous research materials and methods, the main reasons for the inconsistent results may be as follows: different EEG recording methods, and different EEG amplitude and frequency evolution due to different epilepsy periods selected by EEG.

Multivariable analysis based on machine learning provides an opportunity to understand the system by analyzing many features simultaneously [34]. Therefore, we can reduce the I-type error and obtain the optimization model through multivariable analysis. In other words, features extracted from EEG data can be combined with a variety of analyses to develop more reliable and effective models. Previous studies have reported that EEG features can been used to identify a variety of neuropsychiatric diseases, such as epilepsy [23] and schizophrenia [13]. To verify the effect of EEG microstate parameters used for epileptic EEG, we extracted the EEG feature set from EEG microstate series and compared the effect of the EEG feature set with the EEG microstate parameters. The result showed that the classification effect of microstate parameters with an accuracy of 87.18% was better than that of EEG features with an accuracy of 79.55%. Based on the results, we can suggest that the microstate parameters contain information which is difficult to obtain through traditional EEG analysis. This means that microstate parameters can be used as a diagnostic biomarker classifying epilepsy. Compared with previous studies [23,35], there were some limitations in this research: (1) this was a single-center study, and the collection of samples was limited; (2) because we chose the interictal EEG, the results could not represent the EEG signals of epileptic patients; (3) due to the low-quality EEG recording and processing technology, the EEG microstate topographic map that we obtained was not completely consistent with previous research, and there was noise interference, which greatly limits our follow-up research. Despite these limitations, the current results report the microstate changes in epilepsy. Even in the absence of epileptic discharge, the accuracy of the feature prediction of epilepsy reached 87.18%. We anticipate a follow-up study of large-sample, multi-center EEG microstates for epilepsy identification.

## 5. Conclusions

In this study, we explored the possibility of using short-range high-lead EEG signals between seizures for the automatic classification of epilepsy. Therefore, based on microstate analysis technology, we extracted microstate parameters and EEG signal features to classify epileptic EEG signals using the SVM classifier. We found that the microstate parameters (accuracy rate of 89.18%) and EEG features (accuracy rate of 79.55%) extracted from microstate sequences can effectively classify epileptic EEG signals. Moreover, compared with EEG features, microstate parameters can more effectively classify epileptic EEG signals. However, this conclusion has some limitations. We expect that this discovery can be verified by large-sample and multi-center experiments in the future. In addition, we expect to use a variety of classifiers to complete the classification of epileptic EEG to study the impact of classifiers on the intelligent classification of epilepsy.

## Figures and Tables

**Table 1:** Calculation formula linear features.

	Formula
Median	$X_{Me}={\left(\frac{N+1}{2}\right)}^{th}$, *N* is an odd $X_{Me}=\frac{{\left(\frac{N}{2}\right)}^{th}value+{\left(\frac{N+1}{2}\right)}^{th}value}{2}$, *N* is an even
Mean	$\bar{x}=\frac{1}{n}\overset{n}{\underset{i=1}{\sum }}x_{i}$
Skewness	$Skew=\frac{1}{n}\overset{1}{\underset{i=1}{\sum }}{\left(\frac{x_{i}-\bar{x}}{S}\right)}^{3}$
Kurtosis	$Kurt=\frac{1}{n}\overset{1}{\underset{i=1}{\sum }}{\left(\frac{x_{i}-\bar{x}}{S}\right)}^{4}$

**Table 2:** Demographic characteristics of two subject groups. ^a^ means that a *t*-test was used. ^b^ means that the Mann–Whitney U nonparametric test was used.

Group	N	Gender (n) ^b^		Age (Years) ^a^	Education Level ^b^			
		Male	Female	**Mean ± SD**	Primary School	Junior High School	High School	University Graduate
PWEs	27	7	20	26.82±9.66	1	6	7	13
CONs	17	5	12	32.06±11.78	2	1	0	14
*p* value		0.803		0.288	0.084			

**Table 3:** PWE participants’ information.

Number	Gender	Age (Yds)	Course of Epilepsy (Yds)	Abnormal Focus of VEEG
01	Female	40	10	Frontal lobe
02	Male	20	5	Anterior temporal area
03	Female	27	1.5	Anterior temporal area
04	Female	38	34	-
05	Male	20	6	Frontal lobe
06	Female	31	8	Frontal pole
07	Male	19	4	Central frontal area
08	Female	32	8	Middle temporal area
09	Female	44	28	Temporal lobe
10	Female	21	3	Frontal lobe
11	Female	30	4	Anterior temporal area
12	Female	50	7	Anterior temporal area
13	Female	21	3	Anterior temporal area
14	Female	16	1	Frontal lobe
15	Female	20	12	Central frontal area
16	Female	23	3	Anterior temporal area
17	Female	18	10	Right frontal lobe
18	Female	18	9	Frontal lobe
19	Male	40	40	Anterior temporal area
20	Female	18	3.5	Frontal pole
21	Female	18	3.5	Central frontal area
22	Female	32	1	Central frontal area
23	Male	19	11	Frontal lobe
24	Female	23	5	Anterior-middle temporal area
25	Female	15	11	-
26	Male	26	6	Anterior temporal area
27	Male	21	0.5	Anterior-middle temporal area

**Table 4:** EEG microstate parameters (frequency of occurrence, average duration, and proportion of time) and SVM classifier results.

Number	Sub-Band	Accuracy	Recall	Specificity	AUC
1	δ (0.5~4 Hz)	0.5750	0	1	0.6947
2	θ (4~8 Hz)	0.7750	0.5294	0.9565	0.8605
3	α (8~13 Hz)	0.8293	0.5882	1	0.9165
4	β (13~30 Hz)	0.8283	0.5882	1	0.9182
5	γ (30~45 Hz)	0.8718	0.7059	1	0.9452
6	45~80 Hz	0.6571	0.5882	0.7222	0.6508

**Table 5:** The classification results of epilepsy and healthy control subjects by feature set input SVM classifier.

EEG Signal Features Set	Classification Accuracy (%)	Recall (%)	Specificity (%)
Median, second quartile, mean, kurtosis, skewness, fuzzy entropy, PFD, ApEn, SampEn, LZC	79.55	81.84	76.47

## Data Availability

The data are not publicly available due to patients’ privacy protection.

## Abbreviations

VEEG	Video electroencephalogram
EEG	Electroencephalogram
PWEs	Patients with epilepsy
SVM	Support vector machines
PFD	Petrosian fractal dimension
ApEn	Approximate entropy
LZC	Lempel–Ziv complexity
AUC	Area under the curve
ILAE	International League Against Epilepsy
IEDs	Ictal epileptiform discharges
Ms	Millisecond
GFP	Global field power
Hz	Hertz
GMD	Global map dissimilarity
GEV	Global explained variance
TP	True positive
FP	False positive
FN	False negative
TN	True negative
ROC	Receiver operating characteristic
aEEG	Amplitude-integrated electroencephalography
CDSA	Compress spectrum array
